# Simalikalactone D, a Potential Anticancer Compound from *Simarouba tulae*, an Endemic Plant of Puerto Rico

**DOI:** 10.3390/plants9010093

**Published:** 2020-01-11

**Authors:** Belmari Mendez, Jeyshka Reyes, Isabel Conde, Zulma Ramos, Eunice Lozada, Ailed M. Cruz, Gabriela Asencio, Augusto Carvajal, Suranganie Dharmawardhane, Dalice M. Piñero-Cruz, Eliud Hernández, Pablo Vivas, Claudia A. Ospina

**Affiliations:** 1 Natural Sciences Program, University of Puerto Rico at Cayey, Cayey 00736, Puerto Rico; belmari.mendez@upr.edu (B.M.); augustocarvajal@gmail.com (A.C.); 2 Department of Biochemistry, University of Puerto Rico, Medical Sciences Campus, San Juan 00936, Puerto Rico; jeyshka.reyes@upr.edu (J.R.); isabelconde031@gmail.com (I.C.); ailed.cruzcollazo@upr.edu (A.M.C.); su.d@upr.edu (S.D.); pablo.vivas@upr.edu (P.V.); 3 Department of Pharmaceutical Sciences, University of Puerto Rico, School of Pharmacy, San Juan 00936, Puerto Rico; zulma.ramos1@upr.edu (Z.R.); gabriela.asencio1@upr.edu (G.A.); eliud.hernandez@upr.edu (E.H.); 4 Department of Biology, University of Puerto Rico, Río Piedras Campus, San Juan 00936, Puerto Rico; eunice.lozada@upr.edu; 5 Department of Chemistry, University of Puerto Rico, Río Piedras Campus, San Juan 00936, Puerto Rico; dalice.pinero@upr.edu; 6 Department of Chemistry and Physics, Universidad Ana G Mendez, Gurabo 00778, Puerto Rico

**Keywords:** *Simarouba tulae*, quassinoids, simalikalactone D, breast cancer, ovarian cancer, medicinal plants, migration

## Abstract

Species of the genus *Simarouba* have been studied because of their antimalarial and antileukemic activities. A group of oxygenated terpenes called quassinoids have been isolated from species of the *Simarouba* genus, and are responsible for its therapeutic properties. We hypothesized that *Simarouba tulae*, an endemic plant from Puerto Rico, is a natural source rich in quassinoid compounds with anticancer activity. The leaves were processed and extracted with solvents of different polarities. The extracts were screened for their antiproliferative activity, and it was shown that the chloroform extract was the most active extract. This extract was purified using different chromatographic techniques to afford the quassinoid simalikalactone D (SKD). This compound was further characterized using NMR and X-ray diffraction analysis. A reassessment of original structural assignments for SKD is proposed. SKD showed high cytotoxicity activity, with an IC_50_ of 55, 58, and 65 nM in A2780CP20 (ovarian), MDA-MB-435 (breast), and MDA-MB-231 (breast) cell lines, respectively. Exposure to SKD led to 15% inhibition of the migration of MDA-MB-231 cells.

## 1. Introduction

Cancer is a public health problem characterized by the abnormal growth of cells in the body. The World Health Organization (WHO) has estimated that the number of new cancer cases in the world will increase in the next two decades from 18.1 million in 2018 to 29.5 million in 2040 [1]. Therefore, there is a need for more research directed to prevention, treatment, and the discovery of new specific and non-toxic therapeutic agents for this disease. A practical strategy in the drug discovery area is exploring the potential of the chemical constituents isolated from medicinal plants [2,3,4]. 

The overall goal of the present research was to discover and characterize compounds from medicinal plants with potential therapeutic application in the treatment of cancer. Specifically, the anticancer properties of Caribbean plants have been less studied than those of plants from Africa, Asia, and Europe [5]. Since *Simarouba* species are rich in bioactive quassinoid compounds, the main objective was to evaluate the antiproliferative potential of extracts and pure compounds from the leaves of *S. tulae*.

The Simaroubaceae family comprises around 170 species of trees and bushes distributed throughout the tropical regions of America, Asia, Africa, and Australia. This family is characterized by the presence of quassinoids that are responsible for antimalarial, antiviral, insecticide, herbicidal, and antitumor activities [6]. A potent quassinoid named brusatol has been demonstrated to have antileukemic activity with an IC_50_ of ~50 nM [7]. In addition, recent studies have identified brusatol as a unique inhibitor of the Nrf2 pathway, which can selectively reduce the protein levels of Nrf2 via stimulated ubiquitination and proteolysis. As a result, there is interest in the therapeutic potential of brusatol as an agent for overcoming chemoresistance [8,9,10]. All of these reports have produced a particular interest in compounds with the quassin motif in their chemical structure. 

**Figure 1:**
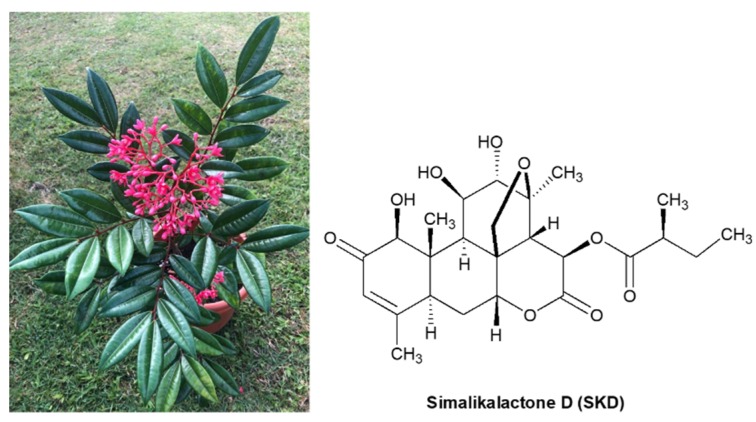
Picture of *Simarouba tulae* plant and structure of similikalactone D (SKD).

In the Antilles, the Simaroubaceae family is represented by the genera *Picrasma*, *Quassia*, and *Simarouba* [11]. *Simarouba tulae* Urb. (“aceitillo tree”) is an endemic species from of Simaroubaceae family from Puerto Rico. *S. tulae* is a tree reported to reach up to 60 feet in height, but overexploitation of its wood and habitat fragmentation has diminished its populations considerably [12]. Today, this species has become a rare species, with a few shrubby 8–15 foot individuals or small trees up to 25 feet in protected forested rainy areas in Puerto Rico, including El Yunque, Carite, Susua, Maricao, and Guilarte Forests. This plant has shiny, dark green, pinnately compound leaves and terminal long-stalked clusters of dark red flowers (Figure 1). 

To our knowledge, no references to the chemical constituents of this species have been reported. In a preliminary study, we investigated the cytotoxic and antiproliferative activity of seven species of Puerto Rican plants, including *Simarouba tulae*, against three breast cancer cell lines. In this study, the crude, hexane, and chloroform extracts from *Simarouba* showed the highest antiproliferative activities in MCF-7 and T47D breast cancer cell lines, inhibiting more than 80% of cell proliferation at concentrations of 100 µg/mL [13]. In this study, leaves of this plant were used to isolate the chemical constituents responsible for its anticancer activity. Thus, this paper describes the bioassay-guided isolation, structural elucidation, revision of original structural assignments, X-ray diffraction analysis, and cytotoxic activities of the quassinoid simalikalactone D (SKD) (Figure 1), a compound previously isolated from *Simaba* and *Quassia* species, which has been recognized to possess anticancer and antimalarial activity [14,15,16]. Our findings revealed that SKD has potent in vitro cytotoxicity, with IC_50_ values of 55 nM in ovarian and from 58 to 67 nM in breast cell lines, including cancer cell lines. This contribution is significant because we have reported the cytotoxic and antimigratory effects of SKD, a quassinoid isolated for the first time from *Simarouba tulae* plant.

## 2. Results and Discussion

### 2.1. Assessment of the Antiproliferative Potential of Extracts in Cancer Cell Lines

#### 2.1.1. Preparation of Plant Extracts

*S. tulae* leaves were collected, dried, and extracted with a 1:1 mixture of dichloromethane–methanol to obtain a crude extract. The resulting crude extract was suspended in water and extracted with solvents of different polarities, including hexane, chloroform, ethyl acetate, and butanol (Table 1). The method for the preparation of the plant extracts of different polarities has been described previously and was conducted as reported [13]. According to our results, the solvents that extracted the greatest amount of metabolites were hexane and chloroform. In addition, ^1^H and ^13^C-NMR analysis of these extracts showed the presence of signals corresponding to highly oxygenated terpenes. 

#### 2.1.2. Determination of the Biological Activity of Plant Extracts in Cancer Cells

Crude mixture, solvent extracts, and collected fractions of *Simarouba* leaves were screened for their antiproliferative activity against three malignant cancer cell lines: MDA-MB-231 (breast), A2780CP20 (cisplatin resistant ovarian), and SH-SY5Y (neuroblastoma) (Table 2 and Table 3). The chloroform extract showed the highest antiproliferative effect against A2780CP20 cells at a concentrations lower than 1 µg/mL. In addition, our NMR analysis of fractions from the chloroform extract showed that Fraction 3 contained the principal constituent of this extract, and thus demonstrated the highest inhibition at a concentration of 44 ng/mL. The chloroform extract also showed antiproliferative activity against MDA-MB-231 cells at a concentration of 22 ng/mL. We also evaluated the antiproliferative activity of *Simarouba* extract on the SH-SY5Y neuroblastoma cancer cell line at a single concentration of 3.125 µg/mL. At this concentration, the chloroform extract, as well as Fraction 2 (SH2C2) and Fraction 3 (SH2C3), showed a percentage of growth inhibition greater than 80%. These results suggest that fractions collected from the chloroform extract contained secondary metabolites with promising anticancer activity. 

### 2.2. Purification, Isolation, and Characterization of Simalikalactone D (SKD)

**Figure 2:**
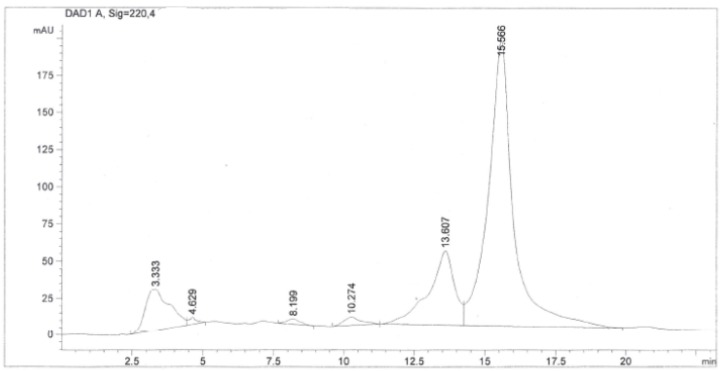
HPLC chromatogram of Fraction 3 (SH2C3) containing simalikalactone D (SKD). SH2C3 fraction was purified on a C18 column with a mixture of methanol and water (55:45, v/v) as a mobile phase, at flow rate of 0.65 mL/min and with UV detection at 220 nm. SKD was collected separately in the interval of 15.57 min.

**Figure 3:**
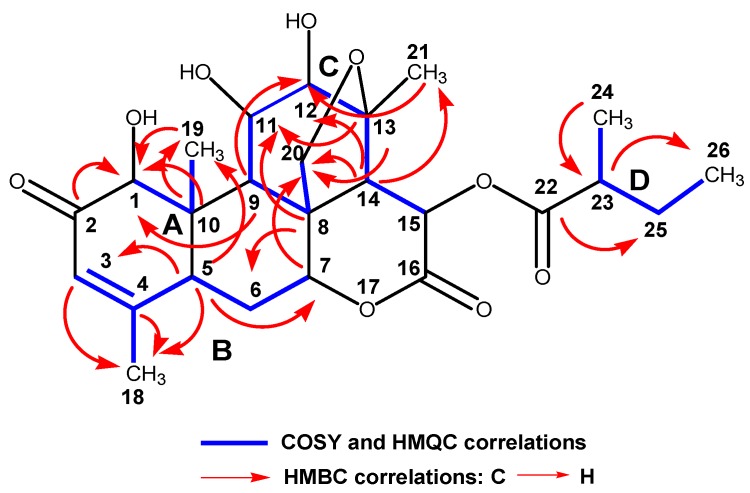
Partial structures for simalikalactone D (SKD) generated from COSY, HMQC, and HMBC experiments.

**Figure 4:**
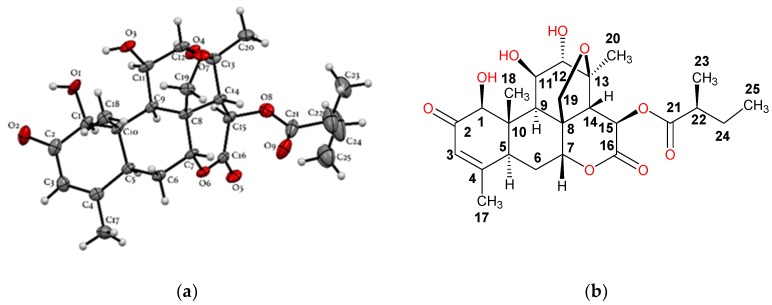
(**a**) Illustration of the crystal structure of SKD with thermal ellipsoids drawn at 50% probability; (**b**) structure of SKD showing all the chiral centers. To facilitate analysis, the carbon numbering system for Positions 17 to 25 are the same as those generated for the X-ray structure (Figure S27, Supplementary Materials).

The chloroform extract (40 g) of *Simarouba tulae* was chromatographed on Si gel with 5% methanol in chloroform to obtain seven fractions (1–7). Fraction 3 (1.1 g) was purified on a sephadex LH-20 column to give six fractions (A–F). Subfraction C (730 mg) was purified successively by column chromatography with a mixture of chloroform/methanol (97:3), and finally by reversed phase HPLC (Figure 2) with a mixture of 45% methanol in water to afford 8 mg of a pure compound which was identified as the known quassinoid simalikalactone D (SKD). In order to obtain greater quantities of SKD, we optimized the purification method. SKD was purified by normal phase column chromatography on silica gel diol using a mixture of chloroform/methanol (97:3) from subfraction D and Fraction 2. Through this purification method, we isolated 150 mg of SKD. The structure of SKD was elucidated by spectroscopic methods and further confirmed by X-ray diffraction. The ^1^H-^1^H COSY, HMQC, and HMBC experiments revealed the presence of four spin systems (A–D) (Figure 3). The spin systems’ connectivity was established unambiguously by HMBC experiment. Systems A and B were confidently linked by the HMBC correlation between C-5 and H_3_-19, whereas systems A and C were connected by the key HMBC correlation between C-9 and H-1. In addition, the HMBC correlation for C-8/H-6b and C-7/H-20a linked systems B and C. Finally, HMBC cross-peaks between C-22 and H-14 allowed the attachment of systems C and D, thus completing the planar structure of SKD. However, our spectroscopic data analysis revealed seven NMR assignments in CDCl_3_ (deuterated chloroform) that were not in accordance with the structure reported in 1993 (Table 4) [14]. Critically, the ^13^C-NMR assignments for quaternary carbons C-8 and C-10 should have been exchanged, as originally reported from HMBC correlations for C-8/H-20b and C-10/H-1/H_3_-19. The same situation of exchange should have been presented for the ^13^C-NMR assignments for carbons C-11 and C-12 from HMBC correlations for C-8/H-11, C-14/H-12, and C-21/H-12. The ^13^C-NMR assignments for methyl groups 19, 21, and 24 should have been revised as originally reported from HMQC correlations with their protons at δ_H_ 1.19, 1.44, and 1.21, respectively. Further analysis of HMBC correlations for C-19 at δ_C_ 11.4 and H-1, C-21 at δ_C_ 22.9 and H-12, and C-24 at δ_C_ 16.6 and H-23, unambiguously established the ^13^C-NMR assignments for C-19, C-21, and C-24. Moreover, the proposed assignments revisions were similar to the assignments reported for other quassinoids isolated from the Simaroubacea family, such as simalikalactone E (SKE) and orinocinolide (Table 4) and to those gathered in a literature review [15,16,17,18]. Thus, the earlier reported NMR assignments for SKD were deemed to be incorrectly positioned, and their δ_C_ was revised as shown in the Materials and Methods section. The relative stereochemistry for the twelve chiral centers in the pentacyclic framework of SKD was established by analysis of NOESY experiment and was similar to the original reported structure [14]. The correlations of H-1/H-5, H-1/H-9, H-9/H-11, and H-9/H-15 suggested that these hydrogens were in α-face. On the other hand, correlations between H-14/H-7, Me-19/H-20b, and H-12/H-20b revealed that these hydrogens were in the β-face. Fortunately, single-crystal X-ray diffraction analysis revealed the stereochemical rearrangements of the 12 chiral centers in SKD through the determination of its relative configuration, as confirmed by NMR experiments (Figure 4). 

### 2.3. Potential Antiproliferative Activity of Simalikalactone D (SKD)

Most scientific studies on SKD have concentrated on developing SKD as an antimalarial agent [16,19,20]. However, there have been some in vitro studies regarding its cytotoxic activities at low concentrations (1.0 to 0.2 µM) in cancer cell lines such as KB (human epidermal carcinoma), P-388 (lymphocytic leukemia), BT-549 (human ductal carcinoma), MCF-7 (breast), SK-OV-3 (ovarian), and Vero cells [14,16,17,21,22]. In this study, we decided to expand the cytotoxic potential of SKD by evaluating its activity in aggressive cancer cell lines associated with a poor prognosis, resistance, and difficult treatment. Therefore, SKD was evaluated for its growth inhibitory potency against several cancer cell lines including A2780CP20 (cisplatin resistant ovarian), MDA-MB-231 (breast), MDA-MB-435 (breast), 4T1 (breast), PC3 (prostate), HCT-116 (colon), and SH-SY5Y (neuroblastoma). As shown in Table 5, SKD showed potent in vitro cytotoxicity, with an IC_50_ of 55 nM against A2780CP20 ovarian cancer cells. In the highly metastatic MDA-MB-231 and MDA-MB-435 cancer cell lines, SKD inhibited cell viability with an IC_50_ of 65 and 58 nM, respectively. However, SKD demonstrated a similar 67 nM IC_50_ in the MCF10A non-cancer mammary epithelial cell line, indicating potential toxic effects in normal breast cells. Additionally, SKD inhibited growth proliferation of the highly aggressive 4T1 mouse mammary tumor cell line with an IC_50_ above 100 nM (IC_50_ = 218 nM). Similarly, in the PC3 prostate cancer, HCT-116 colon cancer, and SH-SY5Y neuroblastoma cell lines, SKD inhibited cell viability with an IC_50_ above 100 nM. Future studies will confirm the cytotoxic potential of SKD in a range of non-cancer cell lines prior to the translational development of SKD for breast and ovarian cancer.

**Figure 5:**
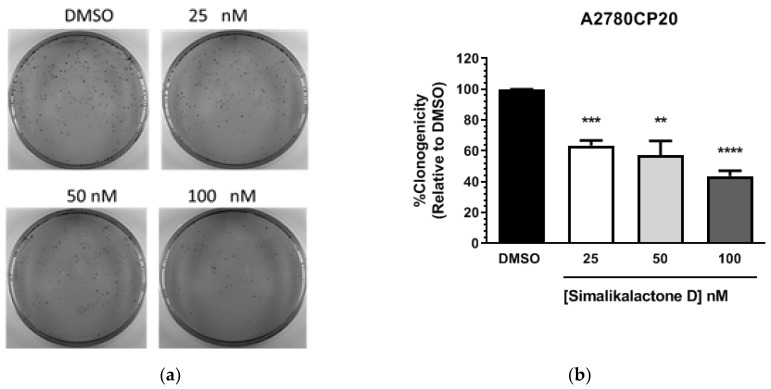
Anti-proliferative effect of SKD in cisplatin-resistant ovarian cancer cells, A2780CP20. Cells were treated with vehicle (DMSO) or SKD. After 24 h, a colony formation assay was performed as described in the Materials and Methods section. (**a**) Representative images of colonies grown in Petri dishes. (**b**) Graph shows that SKD significantly reduced the number of colonies compared with control cultures (100%). Experiments were performed in triplicate. **** *p* < 0.0001, *** *p* < 0.001, **p < 0.01.

We also evaluated the long-term effects of SKD on cell proliferation in cisplatin-resistant ovarian cancer (A2780CP20) cells by performing colony formation assays. Representative Petri dishes for the number of colonies in untreated cells and cells treated with SKD are shown in Figure 5a. Figure 5b shows that SKD significantly reduced the number of colonies compared with untreated cells. Even concentrations as low as 25 nM significantly reduced the number of colonies compared to the DMSO used as a control (Figure 5b). Together, these results indicate that SKD exerts short-term (reduction of cell viability) and long-term effects (colony formation assays) on the growth and proliferation of cancer cells.

### 2.4. Anti-Migratory Activity of Simalikalactone D (SKD)

**Figure 6:**
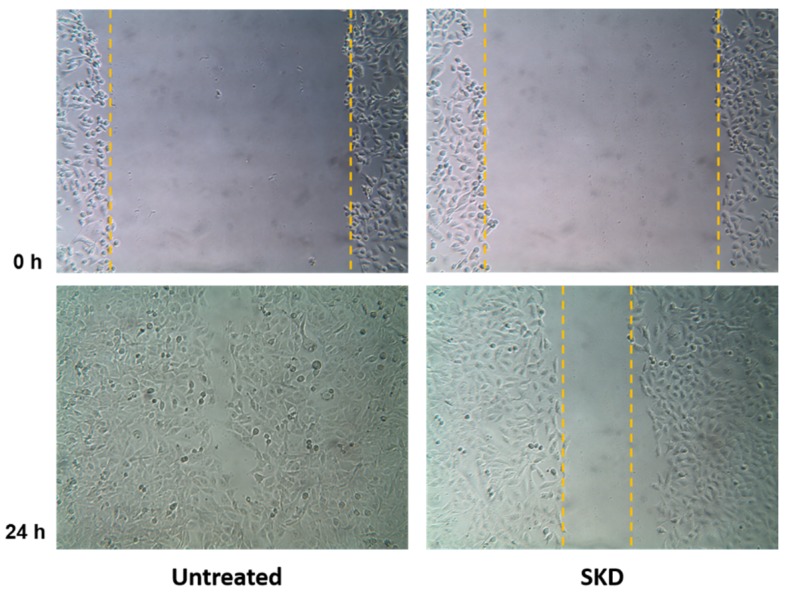
Inhibitory effect of SKD on MDA-MB-231 cell migration detected by a wound-healing assay. MDA-MB-231 were treated with vehicle or with SKD compound. Representative photomicrographs were obtained at 0 and 24 h. Percent relative migration values are the average of three independent experiments. Dotted lines show the area occupied by the initial scraping for 0 h, and the wound edge after 24 h.

The major cause of death in breast cancer patients is the metastasis of primary tumor cells to secondary tissues. To successfully invade a secondary site, a cancer cell completes a series of steps including migration from the primary tumor, invasion of surrounding tissues and basemen membranes, intravasation and survival during circulation, and arrest at a distant target organ [23]. In recent years, many natural products, including compounds from medicinal plants, have been shown to have effective activities against tumor invasion and metastasis [24,25]. The cytotoxic activity of compound SKD on the MDA-MB-231 cell line was determined to be potent, with an IC_50_ of 63 nM using the sulforhodamine B (SRB) assay [26] (Table 5). However, to our knowledge, the anti-migratory activity of SKD remains unexplored. To further assess the anti-migratory activity of SKD in vitro, we examined its inhibitory effects on migration of the metastatic breast cancer cell line MDA-MB-231, chosen due to its enhanced metastatic and migratory properties. The anti-migratory activity was determined using the wound healing assay (scratch assay) [27]. In this assay, the relative migration of MDA-MB-231 cancer cells in the presence of SKD at a concentration of 32 nM (a concentration that did not affect cell viability) was compared to vehicle (0.02% DMSO). Representative micrographs of the migration inhibition of compound SKD are represented in Figure 6. Results show that in the vehicle-treated control experiment, wound healing progressed considerably, and after 24 h, the wound was completely healed. On the other hand, when cells were incubated with SKD after 24 h, wound healing was inhibited by 15%. This low inhibition rate for migration may have been due to our selection of a concentration of SKD that was 50% below the IC_50_ of 65 nM for cell viability inhibition, to avoid confounding effects on cell viability inhibition. 

We will continue with the elucidation and characterization of the molecular mechanisms of SKD in breast and ovarian cancer cell lines, as well as in non-cancer epithelial cells. Potential toxicity in non-cancer cells will be addressed by testing SKD delivery in targeted nanocarriers. We will also improve selectivity by the preparation and isolation of simalikalactone D derivatives. 

## 3. Materials and Methods 

### 3.1. General Experimental Procedures

All reagents and solvents, including dichloromethane, chloroform, methanol, hexane, ethyl acetate, and butanol, were purchased from Sigma Aldrich. The plant extracts were concentrated using a Buchi Rotavapor R-300. Column chromatography was performed using silica gel (35–75 mesh and 200–425 mesh), diol silica gel (75–200 mesh), silica gel C-18 reversed-phase (35–75 mesh), and sephadex LH-20. TLC analyses were carried out using Analtech glass precoated Si gel plates and precoated reversed-phase Si gel plates. Spots were detected on TLC under UV light or iodine vapors chamber. IR spectra were recorded on a Jasco FT/IR 4200 spectrometer. HPLC was performed on a Perkin Elmer Series 200 instrument equiped with a Kromasil C-18 Column (250 × 4.6 mm, 5 μm, flow rate: 1 mL/min). NMR data were recorded on a Bruker NMR spectrometer operating at 400 MHz for ^1^H-NMR and 100 MHz for ^13^C-NMR. All ^1^H-NMR and ^13^C-NMR chemical shifts were referenced to residual CHCl_3_ in the deuterated solvent (7.26 ppm for ^1^H-NMR and 77.0 ppm for ^13^C-NMR). 

### 3.2. Plant Material

Fresh leaves from healthy young adult trees growing in open secondary forest were collected from the Carite State Forest, near the Charco Azul natural swimming pool at Patillas Puerto Rico. The plant species was identified and deposited in the Herbarium at the University of Puerto Rico, Rio Piedras Campus. According to the Holdridge classification system, this is an area of subtropical moist forest, approximately 610 m above sea level (masl) with an annual precipitation of 88.7 inches and annual temperature of 72.3 °F. Soils are well-drained, very deep, clayey soils in the Los Guineos Series [28,29]. Additionally, we obtained vegetative material for *S. tulae* from nursery-grown trees from Reforesta, Inc. San Juan, Puerto Rico.

### 3.3. Spectroscopic Data of Simalikalactone D (SKD)

#### 3.3.1. Simalikalactone D (SKD)

White solid; m.p. 228-231 °C; IR (NaCl) ν_max_ 3442, 3100, 2932, 1744, 1666, 1431, 1379, 1342, 1207, 1145, 1067, 1036, 864, 817 cm^−1^; TLC analysis in CHCl_3_/MeOH (9.7/0.3), R_f_ = 0.37. ^1^H-NMR (CDCl_3_, 400 MHz) δ 6.11 (brs, 1H, H-15), 6.10 (s, 1H, H-3), 4.68 (brs, 1H, H-7), 4.64 (brs, 1H, H-11), 4.63 (d, J = 7.6 Hz, 1H, H-20b), 4.16 (s, 1H, H-1), 3.79 (s, 1H, H-12), 3.54 (d, J = 7.6 Hz, 1H, H-20a), 2.94 (1H, brd, J = 12.8 Hz, 1H, H-5), 2.45 (m, 1H, H-23), 2.43 (m, 1H, H-14), 2.38 (dt, J = 14.5, 2.8 Hz, 1H, H-6b), 2.31 (brs, 1H, H-9), 1.96 (s, 3H, H-18), 1.80 (m, 1H, H6a), 1.75 (m, 1H, H-25b), 1.51 (m, 1H, H-25a), 1.44 (s, 3H, H-21), 1.21 (d, J = 7.0 Hz, 3H, H-24), 1.19 (s, 3H, H-19), 0.98 (t, J = 7.6 Hz, 3H, H-26); ^13^C- NMR (CDCl_3_, 100 MHz) δ 197.2 (C, C-2), 175.4 (C, C-22), 167.7 (C, C-16), 163.5 (C, C-4), 124.2 (CH, C-3), 83.1 (CH, C-7), 81.7 (CH, C-1), 80.4 (C, C-13), 79.4 (CH, C-12), 74.4 (CH, C-11), 71.7 (CH_2_, C-20), 67.3 (CH, C-15), 52.4 (CH, C-14), 47.7 (C, C-10), 45.9 (C, C-8), 43.5 (CH, C-5), 42.5 (CH, C-9), 41.1 (CH, C-23), 28.3 (CH_2_, C-6), 26.5 (CH_2_, C-25), 22.9 (CH_3_, C-21), 22.6 (CH_3_, C-18), 16.6 (CH_3_, C-24), 11.6 (CH_3_, C-26), 11.4 (CH_3_, C-19) (Figures S1–S5, Supplementary Materials). 

#### 3.3.2. Crystal Data for Simalikalactone D (SKD)

SKD was crystallized by slow evaporation of ethyl acetate. X-ray diffraction data were obtained from single crystals mounted on a loop. The data were collected on a Rigaku SuperNova, single-source HyPix3000 diffractometer with Cu Kα radiation (λ = 1.5406 Å) at 100.01(10) K. Data reduction was performed using the program CrysAlis^Pro^. No absorption correction was performed. The structures were resolved through direct method and refined by full-matrix least-square methods on F^2^. All non-hydrogen atoms were refined anisotropically, while H atoms were placed in calculated position with their thermal parameters riding in those of their C atoms (Figure S27, Supplementary Materials). 

### 3.4. Biological Evaluation

#### 3.4.1. Cell Lines and Culture Conditions 

MDA-MB-231, PC3, HCT-116, 4T1, and MCF-10A cells were purcharsed from The American Type of Culture Collection (ATCC). A2780CP20 cells were provided by Dr. Anil K. Sood (MD Anderson Cancer Center). MDA-MB-435 cells were provided by Dr. Suranganie Dharmawardhane (University of Puerto Rico, Medical Sciences) and characterized in Reference [30], from Dr. Danny R. Welch, The University of Kansas Cancer Center, Kansas City, KS. Cells were routinely grown and maintained in RPMI-1640 medium with 10% FBS and with 1% antibiotic/antimicotic solution in a humidified incubator containing 95% air and 5% CO_2_.

#### 3.4.2. Cell Viability and Colony Formation Assays

Cells (3 × 10^4^ cells/mL) were plated in 96 well plates and, 24 h later, serial dilutions (1.0–0.1 mg/mL) of each plant extract in DMSO were added to the cells in triplicate. Blank (medium without cells), experimental (cells following treatment in plant extract), vehicle control (0.1 % DMSO in medium), and positive control (cisplatin) groups were included in each experiment. Seventy-two hours after treatment, the medium was removed and Alamar blue dye (Thermo Fisher Scientific) was added following the manufacturer’s instructions. Optical density (OD) values were obtained using a plate reader (Bio-Rad) and percentages of cell viability were calculated after blank OD subtraction, taking the untreated cell values as 100% cell viability. For assessment of cell proliferation, colony formation assays were performed using Crystal violet dye (Sigma). Briefly, A2780CP20 cells (3 × 10^4^ cells/mL) were seeded into six well plates. Twenty-four hours later, simalikalactone D (SKD) (100, 50, and 25 nM) was added to the cells. The next day, 1000 cells were seeded into 10 cm Petri dishes. Ten days later, colonies were fixed and stained with 0.5% crystal violet solution in methanol. Colonies of at least 50 cells were scored in five random fields using a light microscope (Olympus CKX41) with a total magnification of 40×. Graphs, statistical analysis, and IC_50_ calculations were done using GraphPad Prism (San Diego, CA, USA).

#### 3.4.3. MTT (3-(4,5-Dymethyl thiazol-2-yl)-2,5-diphenyl Tetrazolium Bromide) Assay for Cell Viability

Promega-CellTiter 96^®^ Non-Radioactive Cell Proliferation Assay was performed as per manufacturer’s instructions. Briefly, MCF10A cells were seeded in a 24 well plate and treated for 72 h with a range of SKD concentrations at 0, 16.25 nM, 32.5 nM, 65 nM, 130 nM, and 260 nM in culture medium. After incubation, the MTT reagent was added to the plate (40 μL/well). The plates were incubated for 4 h at 37 °C, stop solution was added to each well, and the plates were incubated to facilitate solubilization of formed formazan salts. The absorbance was measured at 570 nm using a microplate reader. IC_50_ was calculated from sigmoidal dose–response curves using GraphPad Prism V. 6.02, GraphPad Software, Inc. (San Diego, CA, USA).

#### 3.4.4. Sulforhodamine B (SRB) Assay

A stock solution of simalikalactone D (SKD) was prepared at 50 mM in 100% DMSO. For preparation of cells, a flask of 75 cm^2^ or 25 cm^2^ containing 2.6 × 10^5^ cells/mL or 1.44 × 10^5^ cells/mL at 80%–90% confluence was used. Cells were washed with PBS and trypsinized. The concentration of cells was determined from a 1:2 dilution with Trypan Blue, using a hemocytometer. After cells were counted, the concentration was adjusted to 7.0–10.0 × 10^4^ cells/mL. Approximately 100 μL of cell suspension, SKD, and positive and negative controls were added in triplicate to a 96 well plate. Positive control was doxorubicin, and the negative control was DMSO 0.1%. All compounds at concentrations of 50, 25, 12.5, 6.3, 1.6, 0.8, 0.4, 0.2, 0.1, 0.05, and 0.02 μM were incubated with cells at 37 °C for 48 h. For fixation, cold TCA 50% was used and incubated at 4 °C for 1 h. Wells were washed and dried prior to tincture with 100 μL of SRB 0.4 %. To remove excess SRB, acetic acid was used. For analysis, TRIS-BASE solution (pH = 10.5) was used, and shaken prior to quantification using an ELISA reader at 540 nm and SoftMax Pro 4.8 software. For compound SKD, IC_50_ was calculated from sigmoidal dose–response curves (variable-slope) which were generated with data obtained from experiments carried out in triplicate (IC_50_ values were generated with GraphPad Prism V. 6.02, GraphPad Software, Inc.).

#### 3.4.5. Wound Healing Assay (Scratch Assay) Using MDA-MB-231 Cancer Cell Line

Prior to assays, cells were grown to 80%–90% confluence. The cells were washed with PBS to remove all traces of FBS. Cells were detached by incubation in trypsin for 5–10 min at 37 °C. At the end of the incubation time, cells were re-suspended and counted with hemocytometer at a 1:2 dilution with Trypan blue. Subsequently, cell viability was calculated. In a 12 multiwell plate, cells were seeded at 1.5–2.2 × 10^5^ cells/mL in 1 mL and incubated for 24 h. Cells were then rinsed with PBS and incubated in starving media (0.5% FBS) overnight. The compound SKD was tested in triplicate. The negative control for each drug was prepared according to the drug’s DMSO concentration. SKD was diluted and the final concentration in each well was 0.032 µM (or IC_50_/2 on MDA-MB-231 cells). The wound was made using a sterile 200 μL pipette tip. After 24 h incubation, the gap distance was evaluated using Lumera Infinity Analyze 6.4.0 software. Pictures were taken at 0, 8, 12, and 24 h using a 10× objective in an Inverted Laboratory Microscope Leica DM IL LED, and an Infinity1-3 3.1 Megapixel USB 2.0 camera CMOS. The percentage of migration was calculated using the following formulae:

100 − [(X₀/Ẍ₀)] × 100 for time 0 h measurements

100 − [(X₂₄/Ẍ₀)] × 100 for time 24 h measurements

## 4. Conclusions

In this study, we characterized the quassinoid Simalikalactone D (SKD), a metabolite isolated from the Puerto Rican endemic plant *Simarouba tulae*. SKD showed strong cytotoxic activity at nanomolar concentrations against MDA-MB-231 breast, MDA-MB-435 breast, and A2780CP20 ovarian cancer cells. In addition, SKD had antiproliferative effects, as observed from colony formation assays. Moreover, SKD exhibited a migration inhibition of 15% in the metastatic breast cancer line MDA-MB-231. Although SKD inhibited the viability of MDA-MB-231 breast cancer cells, it showed a similar cytotoxic effect on MCF10A mammary epithelial cells. Therefore, future studies will need to determine the usefulness of SKD in adjuvant chemotherapy by delivering SKD in a targeted delivery system to cancer cells. Finally, studies of the mechanism of action focused on the effect of SKD in apoptotic pathways, target identification, and in vivo studies are needed to fully characterize and analyze the potential of this natural product as an anticancer compound.

## Figures and Tables

**Table 1:** Dry Weight of Extracts from *Simarouba tulae* Leaves.

Extract	Dry Weight (g)
Crude ^1^	113
Hexane	31
Chloroform	40
Ethyl acetate	6
Butanol	15

^1^ Crude extract is composed of all chemical compounds.

**Table 2:** Antiproliferative Effect of *Simarouba* Extract/Fraction on Ovarian (A2780CP20) and Breast (MDA-MB-231) Cancer Cell Lines.

Extract/Fraction	A2780CP20 IC_50_ (µg/mL) ^a^	MDA-MB-231 IC_50_ (µg/mL) ^a^
Crude extract	0.75	2.41
Hexane extract	3.67	0.0024
Chloroform extract	0.14	0.0022
Ethyl acetate extract	36.0	N.T.
Butanol extract	0.58	N.T.
SH2C3 fraction ^b^	0.044	N.T.

N.T.: Not tested, ^a^ Generated using the Alamar Blue assay, ^b^ Fraction 3 of chloroform extract. (Figures S6–S13, Supplementary Materials).

**Table 3:** Antiproliferative Effect of *Simarouba* Extract/Fraction on Neuroblastoma SH-SY5Y Cancer Cell Line.

Extract/Fraction	SH-SY5Y %GI ^a^
Crude extract	75
Hexane extract	55
Chloroform extract	83
Ethyl acetate extract	76
Butanol extract	16
SH2C2 fraction ^b^	80
SH2C3 fraction ^b^	88
SH2C4 fraction ^b^	76
SH2C5 fraction ^b^	64

^a^ %GI = percentage of growth inhibition, generated using the sulforhodamine B assay. All extracts were tested at a single dose of 3.125 µg/mL, ^b^ Fractions 2 to 5 (SH2C2 to SH2C5) are from chloroform extract.

**Table 4:** Selected ^1^H NMR and ^13^C NMR Spectral Data for Reported and Revised Simalikalactone D (SKD), Simalikalactone E (SKE), and Orinocinolide.

Atom ^a^	Reported SKD ^b^ δ_H_, δ_C_	Revised SKD ^c^ δ_H_, δ_C_	SKE ^d^ δ_H_, δ_C_	Orinocinolide ^d^ δ_H_, δ_C_
8 ^e^	47.7	45.9	46.1	44.1
10 ^e^	45.9	47.7	50.4	46.5
11	3.77, 79.4	4.64, 74.4	4.75, 74.2	4.70, 74.8
12	4.63, 74.4	3.79, 79.4	3.83, 79.8	3.66, 78.9
19	1.18, 22.9	1.19, 11.4	1.35, 12.5	1.20, 12.0
21	1.43, 16.6	1.44, 22.9	1.45, 22.8	1.39, 23.4
24	1.22, 11.4	1.21,16.6	1.21, 16.7	1.18, 16.7

All spectra were recorded in CDCl_3_, ^a^ The carbon numbering system is in accordance with the numbering of the quassolidane skeleton and the original structure reported for SKD, ^b 1^H-NMR (300 MHz) and ^13^C-NMR (75 MHz), ^c 1^H-NMR (400 MHz), and ^13^C-NMR (100 MHz), ^d 1^H-NMR (500 MHz), and ^13^C-NMR (125 MHz), ^e^ Only **δ_C_** are reported as C-8 and C-10 are quaternary carbons.

**Table 5:** Antiproliferative Effect of SKD on Cell Lines.

Cell Line	IC_50_ (nM) ^a^
A2780CP20 (Ovarian cancer) ^c^	55 ^a^
MDA-MB-231 (Breast cancer) ^d,g^	65 ^a^, 63 ^b^
MDA-MB-435 (Breast cancer) ^d^	58 ^b^
4T1 (Breast cancer) ^e^	>100 ^b^
MCF10A (Breast epithelial cells)	67 ^f^
PC3 (Prostate cancer)	>100 ^a^
HCT-116 (Colon cancer)	>100 ^a^
SH-SY5Y (Neuroblastoma)	>100 (39.8 µM) ^b^

^a^ Generated using the Alamar Blue assay, ^b^ Generated using the sulforhodamine B assay, ^c^ cisplatin-resistant ovarian cancer, ^d^ metastatic mammary adenocarcinoma, ^e^ mouse metastatic mammary carcinoma. ^f^ Generated using the MTT assay, ^g^ IC_50_ of Brusatol using MTT was 390 nM [8] (Figures S14–S26, Supplementary Materials).

## Supplementary Materials

The following are available online at https://www.mdpi.com/2223-7747/9/1/93/s1, Figure S1: ^1^H-NMR Spectrum (400 MHz) of Simalikalactone D (SKD) in CDCl_3_, Figure S2: ^13^C-NMR Spectrum (100 MHz) of Simalikalactone D (SKD) in CDCl_3_, Figure S3: 2D HMQC NMR Spectrum of Simalikalactone D (SKD) in CDCl_3_, Figure S4: 2D COSY NMR Spectrum of Simalikalactone D (SKD) in CDCl_3_, Figure S5: 2D HMBC NMR Spectrum of Simalikalactone D (SKD) in CDCl_3_, Figure S6: Antiproliferative Effect of *Simarouba* Extract/Fraction on A2780CP20 (Ovarian). Figure S7: Antiproliferative Effect of *Simarouba* Hexane Extract/Fraction on A2780CP20 (Ovarian). Figure S8: Antiproliferative Effect of *Simarouba* Chloroform Extract/Fraction on A2780CP20 (Ovarian). Figure S9: Antiproliferative Effect of *Simarouba* Ethyl Acetate Extract/Fraction on A2780CP20 (Ovarian). Figure S10: Antiproliferative Effect of *Simarouba* Butanol Extract/Fraction on A2780CP20 (Ovarian). Figure S11: Antiproliferative Effect of *Simarouba* SH2C3 Fraction on A2780CP20 (Ovarian). Figure S12: Antiproliferative Effect of *Simarouba* Crude Extract on MDA-MB-231 cells. Figure S13: Antiproliferative Effect of *Simarouba* Chloroform Extract on MDA-MB-231 cells. Figure S14: Antiproliferative Effect of SKD on MCF10A cells. Figure S15: Antiproliferative Effect of SKD on MDA-MB-231 cells. Figure S16: Antiproliferative Effect of SKD on SHSY5Y neuroblastoma cells. Figure S17: Antiproliferative Effect of SKD on MDA-MB-435 cells. Figure S18: Antiproliferative Effect of SKD on 4T1 cells. Figure S19: Antiproliferative Effect of SKD on A278CP20 cells. Figure S20: Dose-response data obtained from cell viability of SKD on MCF1A cells. Figure S21: Dose-response data obtained from cell viability of SKD on MDA-MB-231 cells. Figure S22: Dose-response data obtained from cell viability of SKD on SH-SY5Y cells. Figure S23: Dose-response data obtained from cell viability of SKD on MDA-MB-435 cells. Figure S24: Dose-response data obtained from cell viability of SKD on 4T1 cells. Figure S25: Dose-response data obtained from cell viability of SKD on A278CP20 cells. Figure S26: Dose-response data obtained from cell viability of SKD on HCT-116 cells. Figure S27: Illustration of the crystal structure of SKD with H-bond interactions and crystal packing of SKD observed along the b axis. Supporting information regarding the crystal structure in this article can be found in the online version of the journal. Crystallographic information files for SKD is available from the Cambridge Crystallographic Data Center, CCDC 1947777. These data can be obtained free of charge via http://www.ccdc.cam.ac.uk/conts/retrieving.html, or from the Cambridge Crystallographic Data Center, 12 Union Road, Cambridge CB2 1EZ, UK; fax: +44-1223-336-033; or e-mail: deposit@ccdc.cam.ac.uk.
