# Supplementary material for: Simalikalactone D, a Potential Anticancer Compound from Simarouba tulae, an Endemic Plant of Puerto Rico
Source: Plants (Basel). 2020 Jan 11;9(1):93. doi: 10.3390/plants9010093 (PMC7020415; doi:10.3390/plants9010093)
Supplement: Supplementary file 1 [file plants-09-00093-s001.pdf]

Supplementary Material

Simalikalactone D, a potential anticancer compound from *Simarouba tulae*, an endemic plant of Puerto Rico

Belmari Mendez [1], Jeyshka Reyes [2], Isabel Conde [2], Zulma Ramos [3], Eunice Lozada [4], Ailed M. Cruz [2], Augusto Carvajal [1], Suranganie Dharmawardhane [2], Dalice M. Piñero-Cruz [5], Eliud Hernández [3], Pablo Vivas [2] and Claudia A. Ospina [6,*]

[1] Natural Sciences Program, University of Puerto Rico at Cayey, Cayey, PR 00736
[2] Department of Biochemistry, University of Puerto Rico, Medical Sciences Campus, San Juan, PR 00936
[3] Department of Pharmaceutical Sciences, University of Puerto Rico, School of Pharmacy, San Juan, PR 00936
[4] Department of Biology, University of Puerto Rico, Río Piedras Campus, San Juan, PR 00936
[5] Department of Chemistry, University of Puerto Rico, Río Piedras Campus, San Juan, PR 00936
[6] Department of Chemistry and Physics, Universidad Ana G Mendez, Gurabo, PR 00778
* Correspondence: claudiaandreaospina1@gmail.com, ospinac1@uagm.edu; Tel.: +1-787-743-7979, Ext. 4891

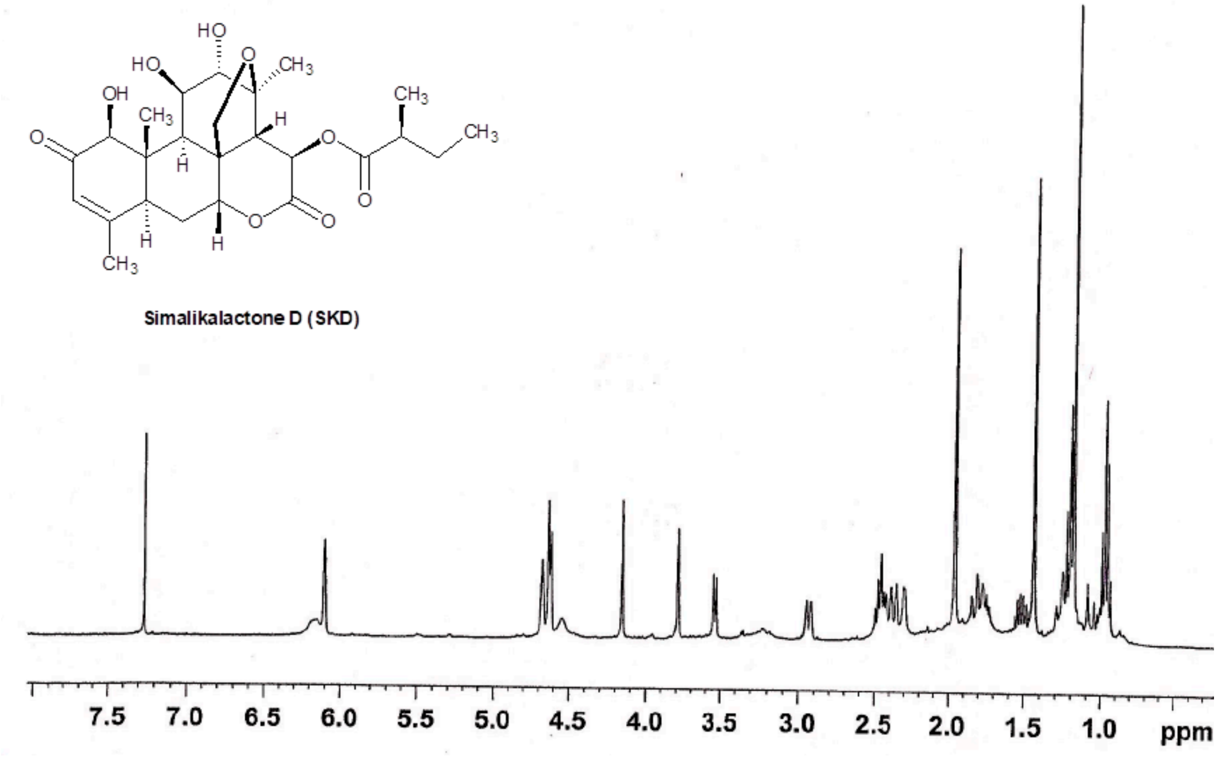

Figure S1: ^{1}H-NMR Spectrum (400 MHz) of Simalikalactone D (SKD) in $CDCl_3$.

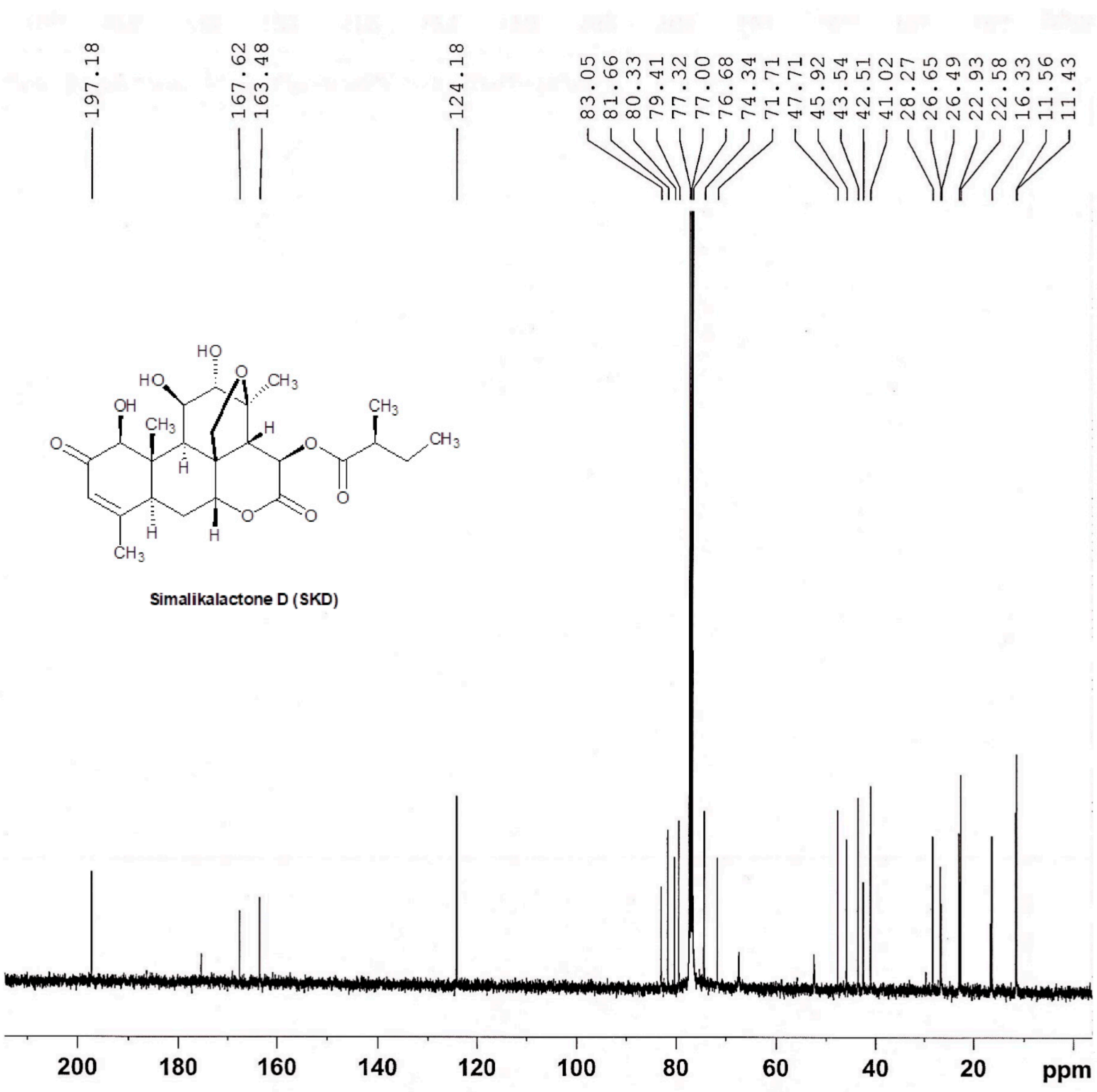

Figure S2: ^{13}C-NMR Spectrum (100 MHz) of Simalikalactone D (SKD) in $CDCl_3$.

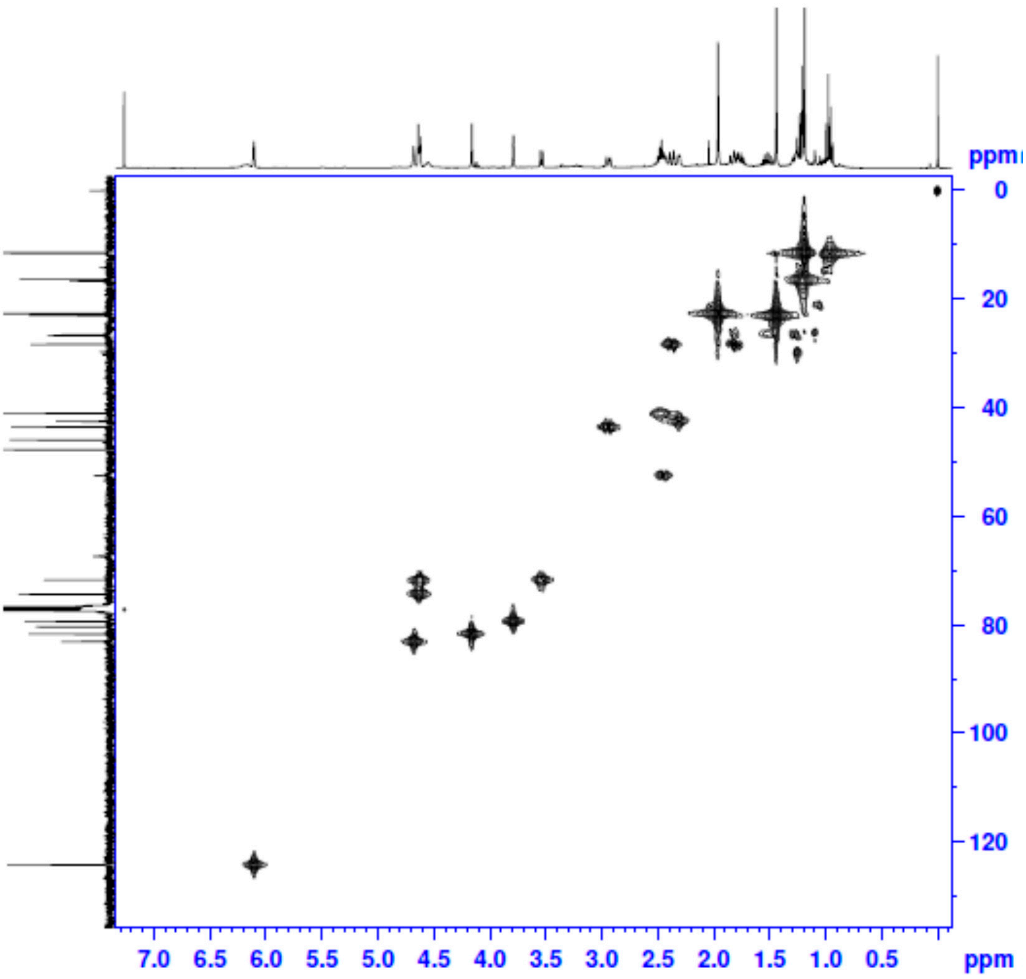

Figure S3: 2D HMQC NMR Spectrum of Simalikalactone D in $CDCl_3$.

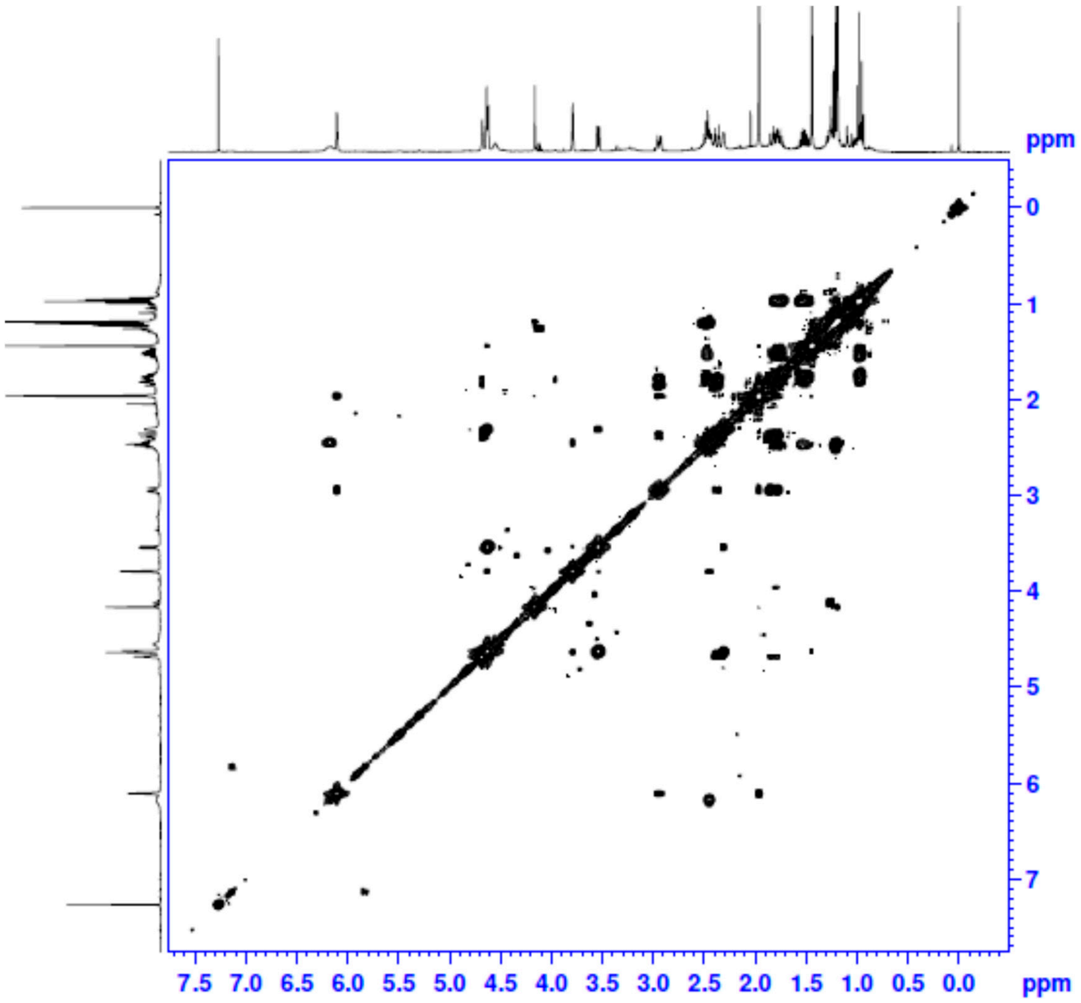

Figure S4: 2D COSY NMR Spectrum of Simalikalactone D in $CDCl_3$.

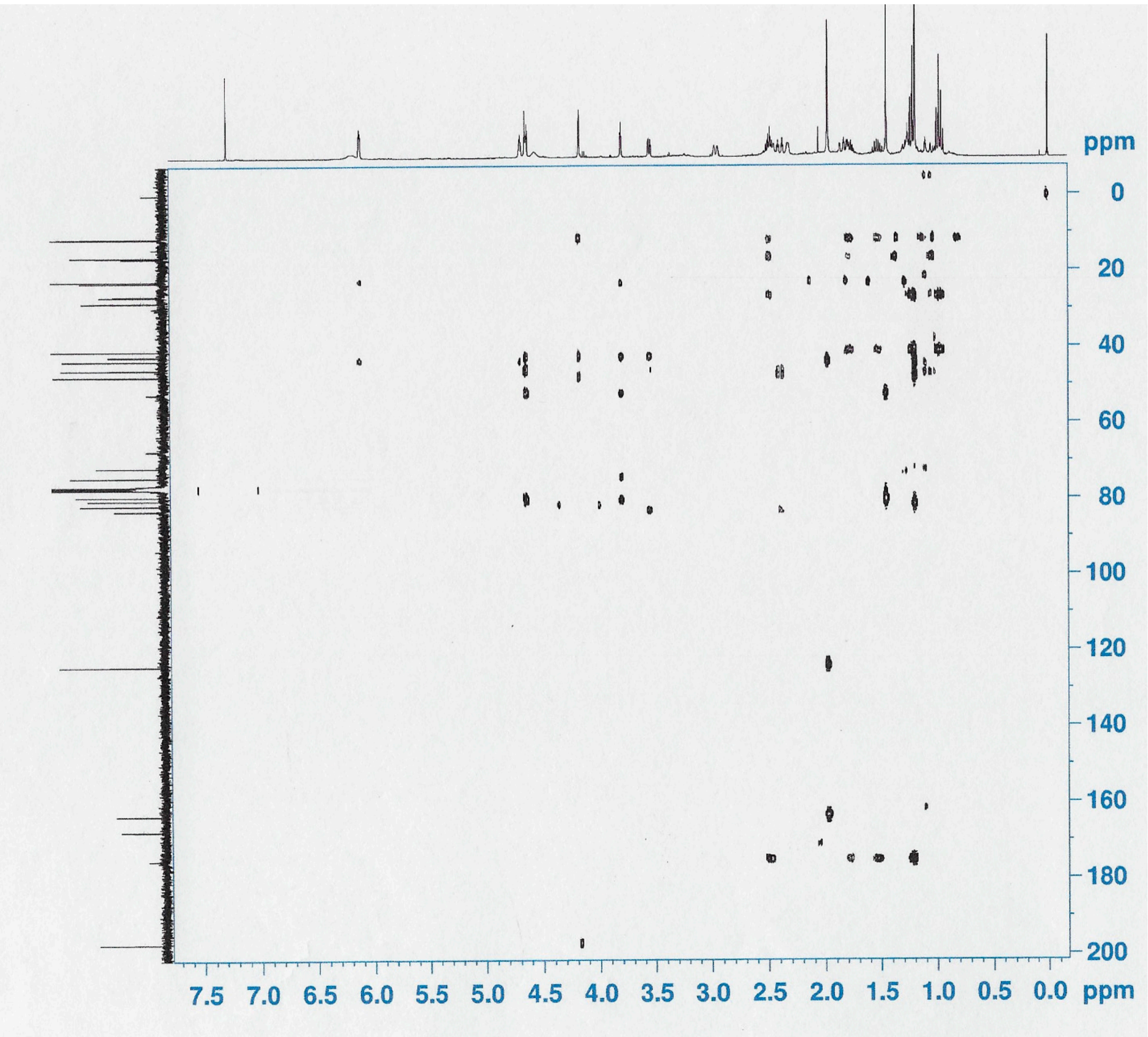

Figure S5: 2D HMBC NMR Spectrum of Simalikalactone D in $CDCl_3$.

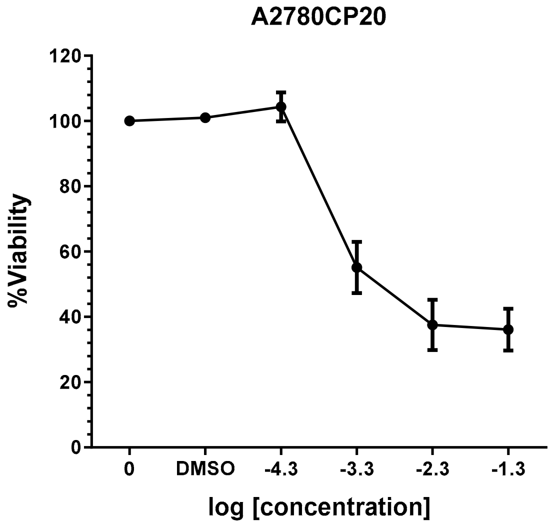

Figure S6: Antiproliferative Effect of Simarouba Extract/Fraction on A2780CP20 (Ovarian).

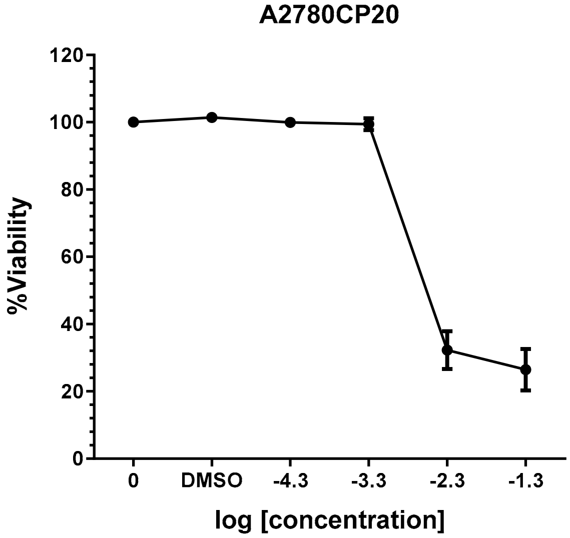

Figure S7: Antiproliferative Effect of Simarouba Hexane Extract/Fraction on A2780CP20 (Ovarian).

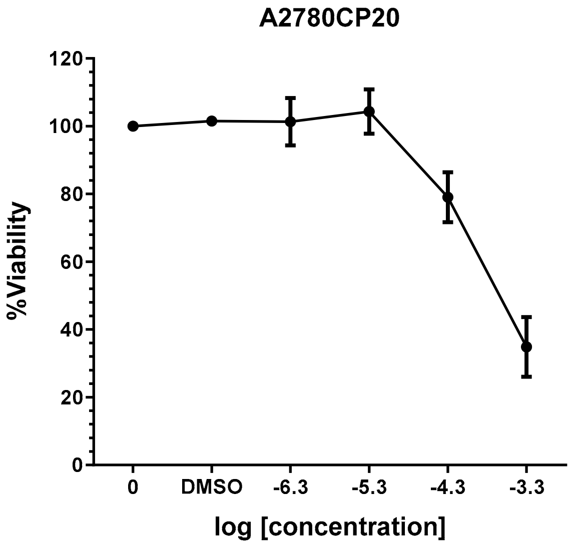

Figure S8: Antiproliferative Effect of Simarouba Chloroform Extract/Fraction on A2780CP20 (Ovarian).

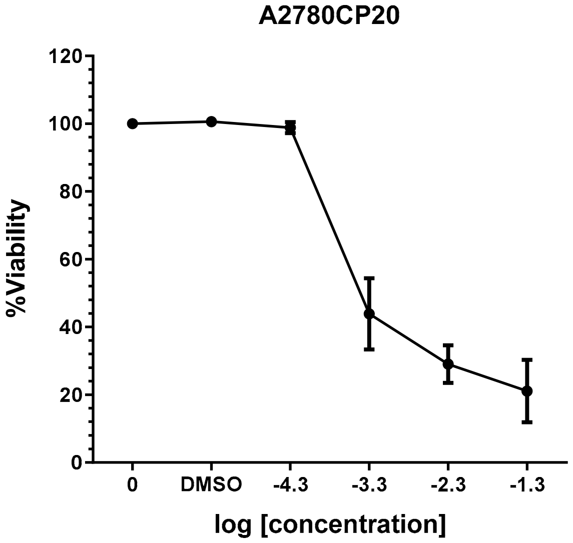

Figure S9: Antiproliferative Effect of Simarouba Ethyl Acetate Extract/Fraction on A2780CP20 (Ovarian).

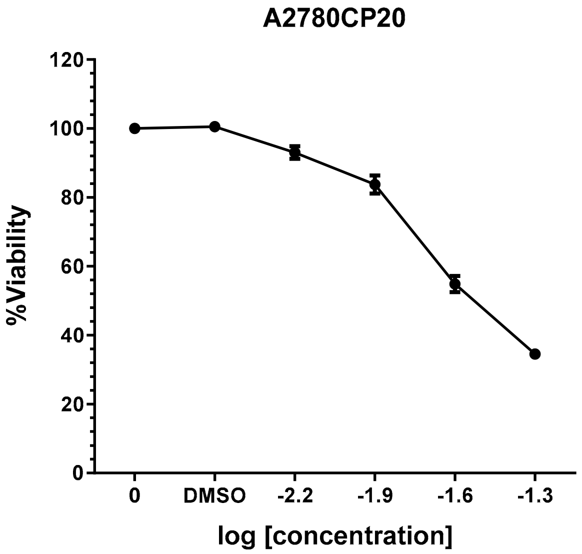

Figure S10: Antiproliferative Effect of Simarouba Butanol Extract/Fraction on A2780CP20 (Ovarian).

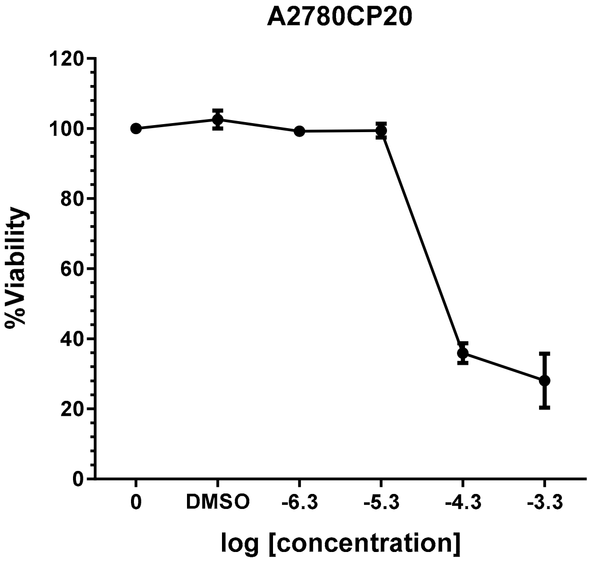

Figure S11: Antiproliferative Effect of Simarouba SH2C3 Fraction on A2780CP20 (Ovarian).

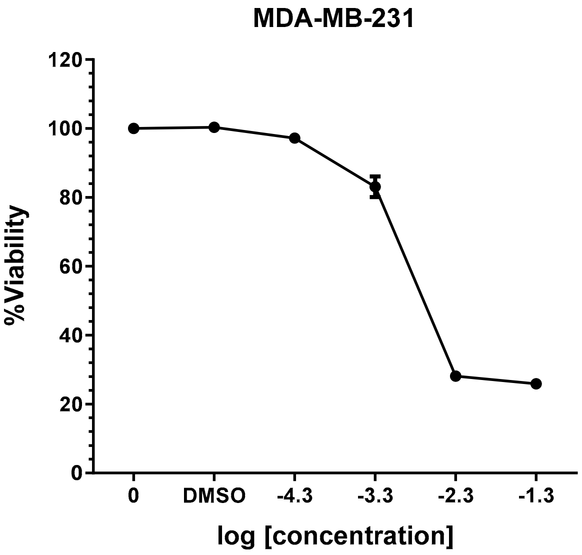

Figure S12: Antiproliferative Effect of Simarouba Crude Extract on MDA-MB-231 cells.

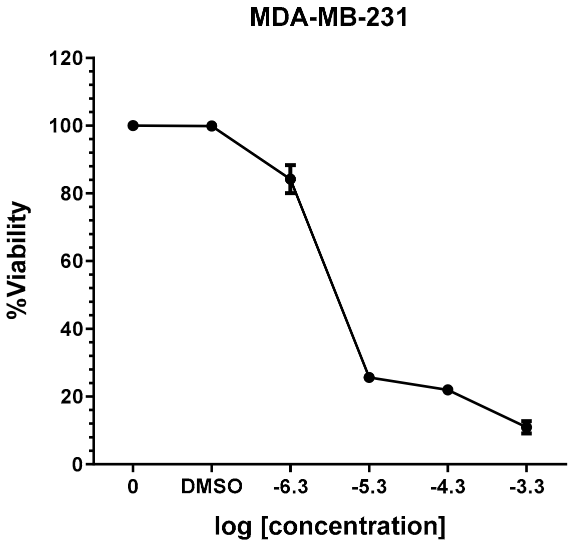

Figure S13: Antiproliferative Effect of Simarouba Chloroform Extract on MDA-MB-231 cells.

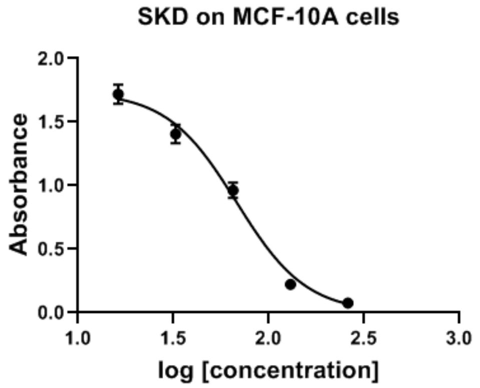

Figure S14: Antiproliferative Effect of Simalikalactone D (SKD) on MCF10A cells.

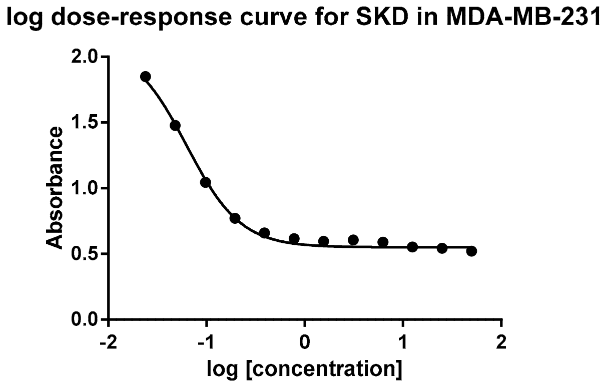

Figure S15: Antiproliferative Effect of SKD on MDA-MB-231 cells.

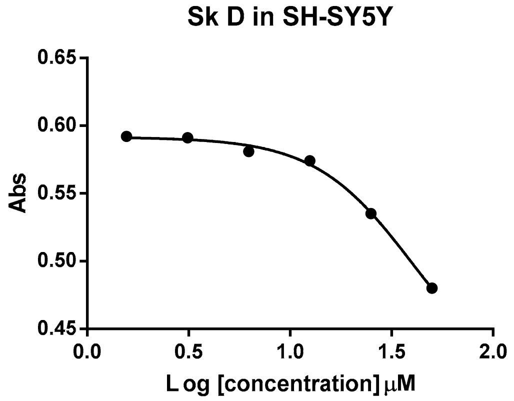

Figure S16: Antiproliferative Effect of SKD on SHSY5Y neuroblastoma cells.

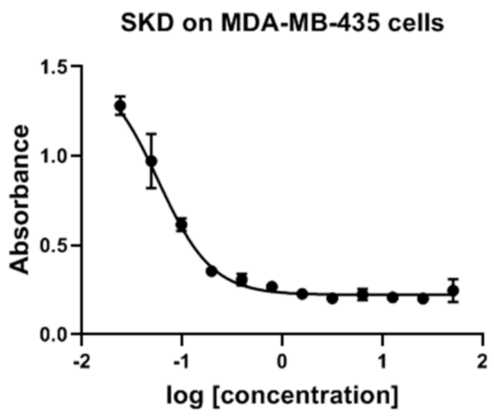

Figure S17: Antiproliferative Effect of SKD on MDA-MB-435 cells.

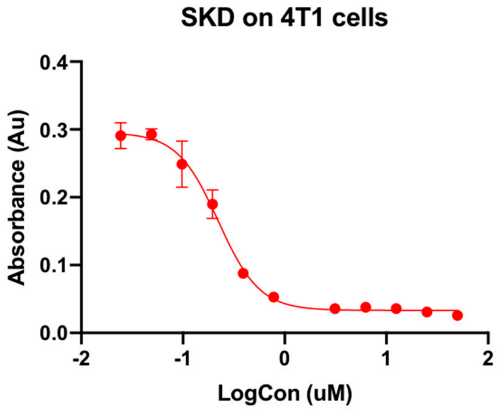

Figure S18: Antiproliferative Effect of SKD on 4T1 cells.

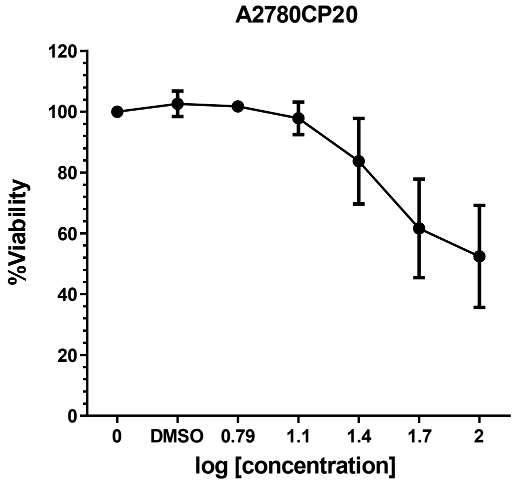

Figure S19: Antiproliferative Effect of SKD on A278CP20 cells.

Conc.				Mean value	SD	SEM	Fold change
NT	2.084	1.798	2.175	2.019	0.197	0.114	1
260 nM	0.075	0.083	0.069	0.076	0.007	0.004	0.037
130 nM	0.202	0.262	0.203	0.222	0.034	0.020	0.110
65 nM	0.919	1.032	0.936	0.962	0.061	0.035	0.477
32.5 nM	1.49	1.351	1.375	1.405	0.074	0.043	0.696
16.25 nM	1.788	1.64	1.729	1.719	0.075	0.043	0.851
[DMSO]	2.077	1.73	1.853	1.887	0.176	0.102	0.934

Figure S20: Dose-response data obtained from cell viability of SKD on MCF1A cells.

Conc.				Mean value	SD
50.000uM	0.496	0.543	0.523	0.521	0.024
25.000uM	0.531	0.541	0.557	0.543	0.013
12.500uM	0.539	0.548	0.568	0.552	0.015
6.250uM	0.560	0.584	0.626	0.590	0.033
3.125uM	0.584	0.596	0.635	0.605	0.027
1.560uM	0.604	0.574	0.609	0.596	0.019
0.780uM	0.599	0.624	0.623	0.615	0.014
0.390uM	0.647	0.664	0.666	0.659	0.010
0.195uM	0.766	0.750	0.797	0.771	0.024
0.098uM	1.032	1.081	1.023	1.045	0.031
0.048uM	1.495	1.483	1.454	1.477	0.021
0.024uM	1.863	1.823	1.860	1.849	0.022

Figure S21: Dose-response data obtained from cell viability of SKD on MDA-MB-231 cells.

Conc.				Mean value	SD
50.000uM	0.550	0.548	0.561	0.553	0.007
25.000uM	0.591	0.574	0.598	0.588	0.012
12.500uM	0.583	0.584	0.601	0.589	0.010
6.250uM	0.617	0.580	0.592	0.596	0.019
3.125uM	0.570	0.575	0.597	0.581	0.014

Figure S22: Dose-response data obtained from cell viability of SKD on SH-SY5Y cells.

Conc.	Mean value	SD
50.000uM	0.247	0.064
25.000uM	0.202	0.020
12.500uM	0.208	0.007
6.250uM	0.224	0.031
3.125uM	0.203	0.011
1.560uM	0.228	0.024
0.780uM	0.268	0.023
0.390uM	0.308	0.031
0.195uM	0.356	0.002
0.098uM	0.616	0.035

Figure S23: Dose-response data obtained from cell viability of SKD on MDA-MB-435 cells.

Conc.	Mean value	SD
50.000uM	0.026	0.004
25.000uM	0.031	0.005
12.500uM	0.036	0.003
6.250uM	0.038	0.003
3.125uM	0.026	0.004
1.560uM	0.031	0.005
0.780uM	0.053	0.005
0.390uM	0.088	0.005
0.195uM	0.190	0.021
0.098uM	0.249	0.034

Figure S24: Dose-response data obtained from cell viability of SKD on 4T1 cells.

Conc.				
0	100	100	100	100
DMSO 0.0001%	100.29	108.62	102.33	99.37
6.25nM	101.43	102.76	103.11	100
12.5nM	89.97	100.69	101.55	99.37
25nM	63.04	86.9	93.52	91.51
50nM	38.97	62.07	76.42	69.18
100nM	30.09	53.1	70.73	55.97

Figure S25: Dose-response data obtained from cell viability of SKD on A278CP20 cells.

Conc.		
0	100	100
DMSO 0.0001%	98.78	102.66
6.25	96.83	103.19
12.5	91.22	97.07
25	88.05	85.9
50	78.78	77.13
100	73.27	67.55

Figure S26: Dose-response data obtained from cell viability of SKD on HCT-116 cells.

Crystallographic Data for SKD (dpc175, CCDC 1947777)

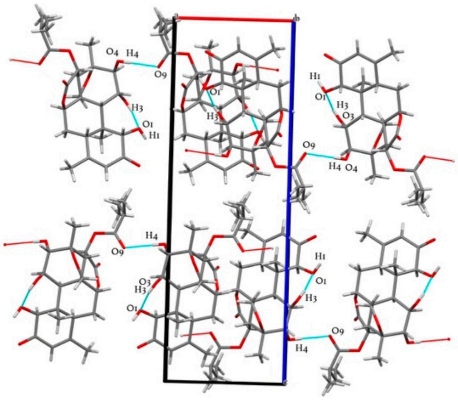

Figure S27: Illustration of the crystal structure of SKD with H-bond interactions and crystal packing of SKD observed along the b axis

Crystal data and structure refinement for dpc175.

Identification code	dpc175
Empirical formula	$C_{25}H_{33}O_9$
Formula weight	477.51
Temperature/K	100.0(3)
Crystal system	orthorhombic
Space group	$P2_12_12_1$
a/Å	8.10370(10)
b/Å	11.70700(10)
c/Å	24.7067(2)
α/°	90
β/°	90
γ/°	90
Volume/Å^3	2343.93(4)
Z	4
ρ_{calc}g/cm^3	1.353
μ/mm^{-1}	0.854
F(000)	1020.0
Crystal size/mm^3	0.188 × 0.128 × 0.037
Radiation	CuKα (λ = 1.54184)
2Θ range for data collection/°	7.156 to 137.522
Index ranges	$-9 \le h \le 9$, $-13 \le k \le 14$, $-29 \le l \le 29$

Reflections collected	37080
Independent reflections	4335 [R_{int} = 0.0726, R_{sigma} = 0.0291]
Data/restraints/parameters	4335/23/316
Goodness-of-fit on F^2	1.044
Final R indexes [I>=2σ (I)]	R_1 = 0.0459, wR_2 = 0.1139
Final R indexes [all data]	R_1 = 0.0478, wR_2 = 0.1155
Largest diff. peak/hole / e Å^{-3}	0.62/-0.52
Flack parameter	0.05(8)

Fractional Atomic Coordinates (×10^4) and Equivalent Isotropic Displacement Parameters (Å^2×10^3) for dpc175. U_{eq} is defined as 1/3 of of the trace of the orthogonalised U_{IJ} tensor.

Atom	*x*	*y*	*z*	U(eq)
O1	7185(3)	4637(2)	1929.7(11)	33.3(6)
O2	7270(3)	4054(3)	911.0(11)	37.9(6)
O3	5905(3)	6106(2)	2693.6(10)	29.4(5)
O4	5290(3)	4076(2)	3766.8(10)	28.9(5)
O5	798(3)	1621.0(19)	3169.8(10)	30.7(6)
O6	771(3)	3030.2(18)	2583.7(9)	23.7(5)
O7	2805(3)	6467.0(18)	3294.1(9)	26.5(5)
O8	1189(3)	3177(2)	4015.7(9)	30.8(6)
O9	-1287(3)	3497(2)	3637.7(13)	42.4(7)
C1	5827(4)	3975(3)	1747.3(14)	25.0(7)
C2	5968(4)	3820(3)	1137.8(14)	28.6(7)
C3	4512(4)	3396(3)	852.6(15)	28.9(8)
C4	3032(4)	3349(3)	1091.0(14)	25.1(7)
C5	2814(4)	3683(3)	1681.9(13)	20.9(6)
C6	1094(4)	4153(3)	1816.9(13)	21.9(7)
C7	872(4)	4244(3)	2423.5(13)	20.1(6)
C8	2239(4)	4851(3)	2735.6(13)	19.6(6)
C9	3974(4)	4465(2)	2541.6(13)	18.9(6)
C10	4159(4)	4498(3)	1906.7(13)	20.3(6)
C11	5386(4)	5008(3)	2879.5(14)	24.3(7)
C12	4973(4)	5119(3)	3483.1(14)	24.6(7)
C13	3158(4)	5446(3)	3598.3(14)	24.1(7)
C14	1948(4)	4614(3)	3341.4(13)	20.0(7)
C15	1940(4)	3363(3)	3489.3(13)	21.1(7)
C16	1081(4)	2615(3)	3075.5(13)	22.3(7)
C17	1567(5)	2879(4)	796.7(16)	37.9(9)

C18	4005(5)	5684(3)	1636.2(14)	26.2(7)
C19	2095(4)	6168(3)	2773.1(13)	24.2(7)
C20	2867(5)	5696(3)	4191.6(14)	29.6(8)
C21	-488(5)	3273(3)	4031.7(18)	38.5(9)
C22	-1239(8)	3077(5)	4586(2)	64.9(16)
C23	-1172(8)	4155(5)	4910(2)	66.1(15)
C24	-724(11)	2020(5)	4830(2)	92(2)
C25	-790(8)	974(5)	4555(2)	63.1(14)

Anisotropic Displacement Parameters (Å^2×10^3) for dpc175. The Anisotropic displacement factor exponent takes the form: $-2\pi^2[h^2a^{*2}U_{11}+2hka^*b^*U_{12}+\ldots]$.

Atom	U_{11}	U_{22}	U_{33}	U_{23}	U_{13}	U_{12}
O1	11.8(11)	47.5(16)	40.5(14)	8.2(12)	-0.3(11)	-5.8(11)
O2	23.3(13)	50.4(17)	40.2(14)	11.6(13)	8.4(11)	5.2(12)
O3	25.4(12)	22.1(12)	40.8(14)	4.5(10)	-3.4(11)	-11.4(10)
O4	17.9(11)	28.0(12)	40.9(14)	10.2(11)	-5.6(10)	0.0(10)
O5	33.2(13)	16.7(11)	42.3(13)	0.1(10)	0.5(11)	-8.2(10)
O6	21.7(11)	18.5(11)	30.9(11)	-1.0(9)	-1.5(10)	-7.2(9)
O7	34.1(13)	12.1(10)	33.4(12)	-0.9(9)	-8.0(11)	-1.6(10)
O8	40.6(15)	22.9(12)	29.1(12)	4.0(10)	-1.3(11)	-2.4(11)
O9	24.2(14)	39.2(15)	63.8(19)	14.1(14)	9.8(13)	2.9(12)
C1	13.8(14)	23.9(16)	37.3(18)	6.2(14)	-0.2(14)	1.4(13)
C2	21.0(16)	25.4(17)	39.3(18)	9.3(14)	4.6(15)	7.7(14)
C3	26.9(18)	29.0(18)	30.8(17)	-0.7(14)	2.2(14)	8.1(15)
C4	23.5(17)	18.0(16)	33.7(17)	0.1(13)	-2.5(14)	3.3(13)
C5	16.2(14)	17.0(14)	29.6(16)	1.2(12)	-0.9(13)	-0.3(12)
C6	15.1(15)	19.5(14)	31.2(16)	-0.8(13)	-4.0(13)	0.8(13)
C7	11.7(13)	16.2(14)	32.5(16)	-0.4(12)	-3.9(13)	-0.3(12)
C8	14.2(14)	12.9(14)	31.9(17)	0.7(12)	-4.6(13)	0.7(12)
C9	12.7(14)	11.9(13)	31.9(16)	3.4(11)	-2.3(13)	0.0(11)
C10	12.5(14)	14.4(14)	34.0(16)	3.7(12)	-1.6(13)	1.4(12)
C11	16.4(15)	17.2(15)	39.2(19)	3.8(13)	-6.6(13)	-3.3(13)
C12	20.2(16)	19.1(16)	34.5(18)	3.3(13)	-9.2(14)	-6.6(13)
C13	25.7(18)	13.7(15)	32.8(17)	2.4(13)	-6.9(14)	-1.2(13)
C14	16.7(15)	14.1(15)	29.0(16)	-1.2(12)	-2.2(13)	0.7(12)
C15	15.1(15)	17.2(16)	31.1(16)	3.3(13)	-2.9(13)	0.4(12)
C16	15.3(14)	19.9(15)	31.6(17)	-0.6(13)	1.9(13)	-3.0(13)
C17	33(2)	43(2)	38(2)	-12.3(17)	-1.8(16)	-3.4(18)
C18	25.8(17)	18.2(16)	34.6(17)	4.5(13)	-3.7(15)	1.1(13)
C19	25.3(16)	16.6(16)	30.8(16)	-1.0(13)	-6.0(14)	4.2(13)

C20	31.4(18)	22.6(17)	34.8(18)	-2.5(14)	-5.9(16)	-3.7(15)
C21	37(2)	25.6(18)	53(2)	-0.5(17)	22.7(19)	-0.5(16)
C22	90(4)	51(3)	54(3)	7(2)	35(3)	5(3)
C23	71(4)	75(3)	52(3)	-18(3)	24(3)	0(3)
C24	149(7)	68(4)	59(3)	22(3)	49(4)	22(4)
C25	81(4)	59(3)	49(3)	12(2)	13(3)	-2(3)

Bond Lengths for dpc175.

Atom	Atom	Length/Å	Atom	Atom	Length/Å
O1	C1	1.419(4)	C5	C10	1.551(4)
O2	C2	1.226(4)	C6	C7	1.513(4)
O3	C11	1.429(4)	C7	C8	1.525(4)
O4	C12	1.431(4)	C8	C9	1.553(4)
O5	C16	1.208(4)	C8	C14	1.540(4)
O6	C7	1.478(4)	C8	C19	1.549(4)
O6	C16	1.333(4)	C9	C10	1.576(4)
O7	C13	1.441(4)	C9	C11	1.553(4)
O7	C19	1.453(4)	C10	C18	1.545(4)
O8	C15	1.452(4)	C11	C12	1.534(5)
O8	C21	1.364(5)	C12	C13	1.546(5)
O9	C21	1.198(5)	C13	C14	1.520(4)
C1	C2	1.521(5)	C13	C20	1.513(5)
C1	C10	1.535(4)	C14	C15	1.510(4)
C2	C3	1.461(5)	C15	C16	1.516(4)
C3	C4	1.337(5)	C21	C22	1.516(6)
C4	C5	1.522(5)	C22	C23	1.495(7)
C4	C17	1.496(5)	C22	C24	1.440(8)
C5	C6	1.535(4)	C24	C25	1.401(8)

Bond Angles for dpc175.

Atom	Atom	Atom	Angle/°	Atom	Atom	Atom	Angle/°
C16	O6	C7	125.8(2)	C5	C10	C9	105.9(2)
C13	O7	C19	109.9(2)	C18	C10	C5	109.9(3)
C21	O8	C15	115.5(3)	C18	C10	C9	116.4(3)
O1	C1	C2	108.7(3)	O3	C11	C9	114.4(3)
O1	C1	C10	112.5(3)	O3	C11	C12	107.5(3)
C2	C1	C10	111.6(3)	C12	C11	C9	113.3(3)
O2	C2	C1	119.4(3)	O4	C12	C11	111.4(3)

O2	C2	C3	123.4(3)	O4	C12	C13	107.0(3)
C3	C2	C1	117.2(3)	C11	C12	C13	114.1(3)
C4	C3	C2	121.7(3)	O7	C13	C12	107.3(3)
C3	C4	C5	121.1(3)	O7	C13	C14	100.7(2)
C3	C4	C17	120.8(3)	O7	C13	C20	108.3(3)
C17	C4	C5	118.0(3)	C14	C13	C12	112.2(3)
C4	C5	C6	113.9(3)	C20	C13	C12	112.0(3)
C4	C5	C10	114.8(3)	C20	C13	C14	115.4(3)
C6	C5	C10	109.9(2)	C13	C14	C8	101.1(2)
C7	C6	C5	110.4(3)	C15	C14	C8	114.2(3)
O6	C7	C6	101.7(2)	C15	C14	C13	121.5(3)
O6	C7	C8	110.6(2)	O8	C15	C14	111.3(3)
C6	C7	C8	116.6(3)	O8	C15	C16	109.0(3)
C7	C8	C9	111.5(3)	C14	C15	C16	113.6(3)
C7	C8	C14	107.3(3)	O5	C16	O6	119.4(3)
C7	C8	C19	116.0(3)	O5	C16	C15	120.9(3)
C14	C8	C9	112.7(2)	O6	C16	C15	119.4(3)
C14	C8	C19	96.3(2)	O7	C19	C8	105.3(2)
C19	C8	C9	112.1(3)	O8	C21	C22	114.5(4)
C8	C9	C10	112.7(2)	O9	C21	O8	122.2(3)
C8	C9	C11	112.5(3)	O9	C21	C22	123.3(4)
C11	C9	C10	117.0(3)	C23	C22	C21	110.0(4)
C1	C10	C5	106.3(2)	C24	C22	C21	113.1(4)
C1	C10	C9	109.2(3)	C24	C22	C23	119.4(5)
C1	C10	C18	108.6(3)	C25	C24	C22	122.5(6)

Hydrogen Bonds for dpc175.

D	H	A	d(D-H)/Å	d(H-A)/Å	d(D-A)/Å	D-H-A/°
O3	H3	O1	0.82	2.12	2.756(4)	134.8
O4	H4	O9[1]	0.82	2.05	2.873(4)	178.8

[1]1+X,+Y,+Z

Torsion Angles for dpc175.

A	B	C	D	Angle/°	A	B	C	D	Angle/°
O1	C1	C2	O2	13.7(4)	C8	C9	C10	C18	63.2(3)
O1	C1	C2	C3	-166.2(3)	C8	C9	C11	O3	-86.3(3)
O1	C1	C10	C5	-179.9(3)	C8	C9	C11	C12	37.4(3)
O1	C1	C10	C9	-66.0(3)	C8	C14	C15	O8	-162.9(3)

O1	C1	C10	C18	61.9(3)	C8	C14	C15	C16	-39.5(4)
O2	C2	C3	C4	-167.5(3)	C9	C8	C14	C13	67.7(3)
O3	C11	C12	O4	-149.2(3)	C9	C8	C14	C15	-64.5(3)
O3	C11	C12	C13	89.6(3)	C9	C8	C19	O7	-82.7(3)
O4	C12	C13	O7	-177.4(2)	C9	C11	C12	O4	83.4(3)
O4	C12	C13	C14	-67.7(3)	C9	C11	C12	C13	-37.8(4)
O4	C12	C13	C20	63.9(3)	C10	C1	C2	O2	138.4(3)
O6	C7	C8	C9	71.5(3)	C10	C1	C2	C3	-41.5(4)
O6	C7	C8	C14	-52.3(3)	C10	C5	C6	C7	-60.6(3)
O6	C7	C8	C19	-158.5(3)	C10	C9	C11	O3	46.6(4)
O7	C13	C14	C8	46.7(3)	C10	C9	C11	C12	170.3(3)
O7	C13	C14	C15	174.3(3)	C11	C9	C10	C1	53.8(3)
O8	C15	C16	O5	-46.0(4)	C11	C9	C10	C5	167.9(2)
O8	C15	C16	O6	140.1(3)	C11	C9	C10	C18	-69.6(4)
O8	C21	C22	C23	83.8(6)	C11	C12	C13	O7	-53.8(3)
O8	C21	C22	C24	-52.5(7)	C11	C12	C13	C14	55.9(4)
O9	C21	C22	C23	-96.0(6)	C11	C12	C13	C20	-172.5(3)
O9	C21	C22	C24	127.7(6)	C12	C13	C14	C8	-67.2(3)
C1	C2	C3	C4	12.4(5)	C12	C13	C14	C15	60.5(4)
C2	C1	C10	C5	57.6(3)	C13	O7	C19	C8	-7.5(4)
C2	C1	C10	C9	171.5(3)	C13	C14	C15	O8	75.5(4)
C2	C1	C10	C18	-60.6(3)	C13	C14	C15	C16	-161.0(3)
C2	C3	C4	C5	-2.9(5)	C14	C8	C9	C10	169.6(2)
C2	C3	C4	C17	-178.2(3)	C14	C8	C9	C11	-55.4(3)
C3	C4	C5	C6	151.2(3)	C14	C8	C19	O7	35.0(3)
C3	C4	C5	C10	23.2(4)	C14	C15	C16	O5	-170.8(3)
C4	C5	C6	C7	168.9(3)	C14	C15	C16	O6	15.4(4)
C4	C5	C10	C1	-49.2(3)	C15	O8	C21	O9	-0.1(5)
C4	C5	C10	C9	-165.3(3)	C15	O8	C21	C22	-179.9(3)
C4	C5	C10	C18	68.1(3)	C16	O6	C7	C6	157.9(3)
C5	C6	C7	O6	-70.5(3)	C16	O6	C7	C8	33.3(4)
C5	C6	C7	C8	50.0(3)	C17	C4	C5	C6	-33.5(4)
C6	C5	C10	C1	-179.2(3)	C17	C4	C5	C10	-161.5(3)
C6	C5	C10	C9	64.6(3)	C19	O7	C13	C12	93.4(3)
C6	C5	C10	C18	-61.9(3)	C19	O7	C13	C14	-24.2(3)
C6	C7	C8	C9	-44.1(3)	C19	O7	C13	C20	-145.6(3)
C6	C7	C8	C14	-167.9(3)	C19	C8	C9	C10	-83.1(3)
C6	C7	C8	C19	85.9(3)	C19	C8	C9	C11	51.9(3)
C7	O6	C16	O5	172.7(3)	C19	C8	C14	C13	-49.4(3)
C7	O6	C16	C15	-13.4(4)	C19	C8	C14	C15	178.3(3)
C7	C8	C9	C10	48.9(3)	C20	C13	C14	C8	163.0(3)
C7	C8	C9	C11	-176.1(3)	C20	C13	C14	C15	-69.4(4)

C7	C8	C14	C13	-169.2(2)	C21	O8	C15	C14	74.4(4)
C7	C8	C14	C15	58.6(3)	C21	O8	C15	C16	-51.6(4)
C7	C8	C19	O7	147.7(3)	C21	C22	C24	C25	-49.7(10)
C8	C9	C10	C1	-173.4(2)	C23	C22	C24	C25	178.5(6)
C8	C9	C10	C5	-59.3(3)					

Hydrogen Atom Coordinates (Å×10^4) and Isotropic Displacement Parameters (Å^2×10^3) for dpc175.

Atom	*x*	*y*	*z*	U(eq)
H1	7742.55	4835.69	1669.37	50
H3	6494.05	6030.45	2426.05	44
H4	6266.94	3906	3734.34	43
H1A	5904.72	3218.64	1915.72	30
H3A	4616.74	3153.35	495.92	35
H5	2922.87	2973.06	1888.81	25
H6A	958.16	4900.01	1653.41	26
H6B	257.22	3649.61	1668.34	26
H7	-184.58	4617.78	2500.78	24
H9	4020.13	3650.39	2632.02	23
H11	6340.57	4497.26	2851.14	29
H12	5681.48	5713.78	3637.32	30
H14	842.16	4903.81	3424.36	24
H15	3090.81	3110.6	3512.25	25
H17A	1119.73	2247.42	996.19	57
H17B	1899.1	2625.01	443.7	57
H17C	743.92	3464.04	761.48	57
H18A	2943.33	6004.21	1717.19	39
H18B	4123.37	5604.43	1251.44	39
H18C	4854	6178.88	1772.1	39
H19A	2699.86	6530.19	2481.11	29
H19B	949.56	6404.24	2753.96	29
H20A	3578.15	6307.14	4304.77	44
H20B	3102.68	5025.22	4401.31	44
H20C	1737.46	5914.86	4245.14	44
H23A	-1717.07	4756.16	4715.39	99
H23B	-41.86	4362.35	4972.06	99
H23C	-1715.12	4038.32	5251.03	99
H24A	411.22	2124.8	4944.16	110
H24B	-1372.13	1928.6	5157.69	110
H25A	-312.89	1060.72	4202.21	95

H25B	-1918.93	736.18	4520.75	95
H25C	-184.16	409.38	4755	95

Crystal structure determination of [dpc175]

Crystal Data for $C_{25}H_{33}O_9$ (M =477.51 g/mol): orthorhombic, space group $P2_12_12_1$ (no. 19), a = 8.10370(10) Å, b = 11.70700(10) Å, c = 24.7067(2) Å, V = 2343.93(4) Å^3, Z = 4, T = 100.0(3) K, μ(CuKα) = 0.854 mm^{-1}, $Dcalc$ = 1.353 g/cm^3, 37080 reflections measured (7.156° ≤ 2Θ ≤ 137.522°), 4335 unique (R_{int} = 0.0726, R_{sigma} = 0.0291) which were used in all calculations. The final R_1 was 0.0459 (I > 2σ(I)) and wR_2 was 0.1155 (all data).

Refinement model description

Number of restraints - 23, number of constraints - unknown.
Details:

```
1. Fixed Uiso
 At 1.2 times of:
  All C(H) groups, All C(H,H) groups
 At 1.5 times of:
  All C(H,H,H) groups, All O(H) groups
2. Rigid bond restraints
 C22, C23, C24, C25
 with sigma for 1-2 distances of 0.01 and sigma for 1-3 distances of 0.01
3. Uiso/Uaniso restraints and constraints
C22 ≈ C23 ≈ C24 ≈ C25: within 2A with sigma of 0.04 and sigma for
terminal atoms of 0.08
4.a Ternary CH refined with riding coordinates:
 C1(H1A), C5(H5), C7(H7), C9(H9), C11(H11), C12(H12), C14(H14), C15(H15)
4.b Secondary CH2 refined with riding coordinates:
 C6(H6A,H6B), C19(H19A,H19B), C24(H24A,H24B)
4.c Aromatic/amide H refined with riding coordinates:
 C3(H3A)
4.d Idealised Me refined as rotating group:
 C17(H17A,H17B,H17C), C18(H18A,H18B,H18C), C20(H20A,H20B,H20C), C23(H23A,H23B,
 H23C), C25(H25A,H25B,H25C)
4.e Idealised tetrahedral OH refined as rotating group:
 O1(H1), O3(H3), O4(H4)
```

This report has been created with Olex2, compiled on May 18 2018 14:05:52 for OlexSys.
